# Toward a Contextually Valid Assessment of Partner Violence: Development and Psycho-Sociometric Evaluation of the Gendered Violence in Partnerships Scale (GVPS)

**DOI:** 10.3389/fpsyg.2020.607671

**Published:** 2021-01-11

**Authors:** Katharina Goessmann, Hawkar Ibrahim, Laura B. Saupe, Frank Neuner

**Affiliations:** ^1^Department of Clinical Psychology and Psychotherapy, Bielefeld University, Bielefeld, Germany; ^2^Department of Clinical Psychology, Koya University, Koya, Iraq; ^3^Vivo International, Konstanz, Germany; ^4^Department of Clinical and Biological Psychology, Catholic University of Eichstätt-Ingolstadt, Eichstätt, Germany

**Keywords:** violence against women, partner violence, scale development, violence, assessment, Middle East

## Abstract

This article presents a new measure for intimate partner violence (IPV), the Gendered Violence in Partnerships Scale (GVPS). The scale was developed in the Middle East with the aim to contribute to the global perspective on IPV by providing a contextual assessment tool for partner violence against women in violent-torn settings embedded in a patriarchal social structure. In an effort to generate a scale including IPV items relevant to the women of the population, a pragmatic step-wise procedure, with focus group discussions and expert panels, was performed. The study’s analyses resulted in an 18-item checklist featuring four subscales of the GVPS that are based on a new typology of male-to-female partner violence presenting an alternative to the commonly used classification by type of abuse (i.e., physical, psychological, sexual acts). Therein, dominating behaviors, existential threats, impulsive aggression, and aggravated physical assault were identified as reflective of the lived realities of women in the war-torn Middle East, which was confirmed in factor analysis. The scale’s psychometric properties were assessed with data from 1,009 displaced women in Iraq, and associations with measures of psychopathology were determined. Implications for IPV assessment and prevention possibilities in humanitarian contexts and beyond are discussed.

## Introduction

Intimate partner violence (IPV) against women is an ongoing global human rights issue that brings about a wide range of devastating effects on the health and wellbeing of individuals as well as societies at large (Heise and García-Moreno, 2002; Bonomi et al., 2006; Ellsberg and Emmelin, 2014). IPV is a multifaceted phenomenon that can manifest in a myriad of often co-occurring forms, including physical, verbal, and sexual behaviors. It occurs across all social, religious, and cultural contexts (Krane, 1996; Ellsberg et al., 2015), with 30% of all women worldwide reporting having experienced physical or sexual forms of IPV during their lifetime (Devries et al., 2013).

Studying the global prevalence, impact, and conditions of IPV is a difficult task that involves several challenges and requires thoughtful ethical considerations (Bender, 2017). Although IPV continues to affect many women across the world, it is typically considered a private issue and often remains hidden from direct observation. Many affected women fear negative consequences when reporting experiences of partner violence (Krane, 1996; Pournaghash-Tehrani, 2011). Stigmatization and victim-blaming due to socially embedded gender inequality and inadequate support systems seem also to hinder the reporting of IPV incidents (Overstreet et al., 2019). Given such challenges, underreporting is likely, and getting help is difficult for many affected women worldwide.

Still, existing prevalence research indicates alarming rates of IPV particularly in low and middle-income countries, with prevalence rates between 35 and 66% reported in countries in South Asia, Andean South America, Oceania, most parts of Africa, and the Middle East (Devries et al., 2013). One possible explanation for these high numbers may be related to intertwined impacts of factors identified as increasing the risk of violence against women, such as poverty and gender inequalities (Ellsberg et al., 2015; Heise and Kotsadam, 2015; González and Rodríguez-Planas, 2020). IPV seems to be influenced by social and cultural factors, since conditions like high gender inequality and economic dependence are associated with increased levels of oppression and violence against women (Jewkes, 2002; Ebbeler et al., 2017). Besides, in some geographical regions, the high prevalence rates may be further explained by armed conflicts and subsequent social instabilities, which have been associated with violence against women on several, including interpersonal, levels (Catani, 2010; Stark and Ager, 2011). Research indicates that rates of domestic violence against women increase when men seek to reassert power and reestablish their dominant gender roles when such roles are challenged by war or post-war living conditions (DeLargy, 2013; Guruge et al., 2017; MacKenzie and Foster, 2017).

A contextually valid assessment of IPV in diverse contexts is challenged by the fact that existing instruments have predominantly been developed and validated within relatively stable European or United States-American populations. Those instruments are often exhaustively used without or with limited cultural adaptations, which bears the risk of hiding potential context-specific phenomena and relationships (Haddad et al., 2011; Amawi et al., 2014; Wangel and Ouis, 2019). Furthermore, with most of the existing prevalence research still conducted in relatively stable Western countries, population-based data of IPV from other geographical regions, especially from more fragile (e.g., conflict-torn) societies, remain scarce (Falb et al., 2015; Heise and Kotsadam, 2015). The paucity of IPV assessment and instrument development in non-Western contexts and an inadequate variety of items call for a local development and extensive empirical validation of instruments. The comprehensive understanding of IPV globally requires the rigorous investigation of violence in a variety of contexts, including in unstable and violence-affected populations where IPV rates are reportedly high (Stark and Ager, 2011), and where the complexity of the occurrence of IPV may be influenced by several individual and structural factors (Pournaghash-Tehrani, 2011; Jayasundara et al., 2014; Wachter et al., 2018; Goessmann et al., 2019). Local pragmatic approaches are required in order to perform assessments of IPV which adequately reflect the experiences of women within their social environments. As recommended by researchers and practitioners in the field, the involvement of local communities in the definition and development process is crucial to this in order to reduce power imbalances between researchers and participants in women and violence research (Webb, 1993; Hossain and McAlpine, 2017; Fineran and Kohli, 2020). Thus, the present development study followed a pragmatic approach in which the inclusion of IPV items relevant to the lived experiences of women was paramount.

While the acts of violence perpetrated against women in heterosexual partnerships may be as diverse as the partnerships themselves, their underlying dynamics are often quite similar with aggressions mostly being used to exert physical, emotional, psychological or economic control over the partner (DeKeseredy, 2011; Devries et al., 2013). Those dynamics are usually connected to larger societal factors, such as the still widespread inequality between men and women under patriarchal order, of which IPV can be both a reflection and a constituent (Heise, 1998; Fulu and Miedema, 2015). However, the gender-related aspects of violence against women in partnerships have largely been ignored in existing IPV measurement research (Reed, 2008; Hamby, 2014; Ali et al., 2016; Bender, 2017). Violence against women, including partner violence against women, is per definition any act directed against women because they are women, or that affects women disproportionally (OHCHR, 1992). Consequently, many types of IPV are inherently gendered, such as sexual acts (e.g., forced penetrative intercourse, forced impregnation) or controlling and coercing behaviors, which make a woman dependent on their male partner and reduce their autonomy.

However, existing assessment tools of IPV usually don’t reflect underlying gendered dynamics. The majority of IPV instruments apply a descriptive, tripartite categorization based on the mere appearance of the violent act, classifying partner violence as either physical, psychological/emotional, or sexual (Gómez-Fernández et al., 2019). While this typology attempts to assess all manifestations of violence, it has drawn criticism in recent years for its contribution to overlooking the gendered nature of IPV against women (Reed, 2008; Reed et al., 2010; DeKeseredy, 2011; Hamby, 2014; Ali et al., 2016; Bender, 2017). In an effort to complement the descriptive categorization of physical, psychological or sexual IPV, a growing body of theory has proposed the use of alternative categories and the inclusion of a greater variety of violent acts (Johnson and Ferraro, 2000; Johnson and Leone, 2005; Kelly and Johnson, 2008; Ali et al., 2016; Velonis, 2016; Mennicke, 2019). Researchers have suggested distinguishing acts of IPV, for example, according to violence severity and intensity, situational influences, perpetrator’s motivations, societal patterns of gender-related dominance/control, and the impacts and personal meanings of the abuse for both the perpetrator and the victim, to allow valid IPV assessment that takes the context of the violence into account (Johnson and Ferraro, 2000; Bogat et al., 2005; Ali et al., 2016; Bender, 2017). Accordingly, a number of other categories and patterns of violent acts against women in partnerships have been identified. While an extensive review of the growing literature in this regard is not feasible within the frame of this study, some theoretical developments should be mentioned. For example, research has distinguished acts that are motivated by the aim to control or dominate the women (Strauchler et al., 2004; Ali et al., 2016). Such acts have been reported to be prevalent in women’s lived partnership experiences especially, but not only, in settings with pronounced patriarchal society structures subordinating women (Felson and Messner, 2000). Examples of controlling IPV may include following the partner around, determining their clothing, limiting their social interactions, as well as reproductive coercion. Other IPV events, such as manipulation and economic threats like being denied financial means, may jeopardize the partner’s sense of personal safety and potential for self-sufficiency (Adams et al., 2008; Voth Schrag, 2015). Such acts are often not explicitly considered in IPV research, and thus remain a largely invisible facet of partner violence (Postmus et al., 2020).

Yet other acts of partner violence may be of rather impulsive types. For example in already ongoing conflict situations, verbal aggression (e.g., yelling, calling names) is likely to be followed by, or simultaneously occurring with, physical violence such as hitting or throwing things (Winstok and Smadar-Dror, 2018). Therein, aggression levels, conflict management styles, or substance abuse (e.g., alcohol) may have important impacts on the level of physical and verbal violence used impulsively within heterosexual partnerships (Derefinko et al., 2011; Graham et al., 2011; Cascardi et al., 2018). Regarding physically violent behaviors, research has distinguished a category of highly intense physical attacks, such as attacks with weapons or fire, which can be extremely harmful and even fatal (WHO, 2005; Stark, 2010). For such acts, a pronounced gender pattern has been identified which suggests that women are much more frequently victimized by severe physical violence than are men (Hamby, 2005; Ansara and Hindin, 2010).

The present study reports the development process of a new IPV event checklist from its initial efforts to empirical testing among displaced Syrian and Iraqi women in northern Iraq. We purposefully chose the study’s location for several reasons. In Iraq, a country with comparatively high structural gender inequalities (World Bank Group, 2019), legislation granting equal rights to women and men is reportedly deficient and not implemented consistently; thus, society-wide human rights violations against women are prevalent (Davis, 2016). That includes the Kurdistan region of Iraq (KRI), where a recent study showed that women in Erbil had little knowledge of existing law enforcement structures and were reluctant to seek justice in cases of domestic violence (Malik et al., 2017). Furthermore, acceptance of physical violence against women seems to be widespread, as 63% of women participating in a survey study conducted in Iraq indicated that they approved of the use of beatings in partnerships (Linos et al., 2012). Despite its comparatively high IPV levels, the Middle East is among the regions for which very few validated IPV instruments exist (Boy and Kulczycki, 2008; Azadarmaki et al., 2016), one of the few exceptions is the Arab version of the Composite Abuse Scale (Alhabib et al., 2013). Hence, additional IPV instruments are necessary, which consider the full variety of experiences of women living in the context. The decision to use data from forcibly displaced women for the development of this new IPV scale based on the specific characteristics of these women. As mentioned earlier, armed conflicts seem to increase rates of interpersonal violence, including violence against women (Catani, 2010; Stark and Ager, 2011; Wachter et al., 2018). In various post-war and displacement settings, particularly high IPV rates are reported (e.g., Ward, 2002; Annan and Brier, 2010; Clark et al., 2010; Jewkes et al., 2017), including in Iraq (Goessmann et al., 2019). Refugee camps in the war-torn KRI thus provide a suitable environment in which to gather data on IPV exposure and to validate a new instrument for its assessment.

## Materials and Methods

The present study is part of a collaborative research project conducted by the University of Bielefeld, Germany, and Koya University, Iraq, which aims to investigate the experiences and psychological states of refugees and forcibly displaced people living in camps in the KRI. The project and its procedures featuring Syrian and Iraqi individuals and married couples have been approved by the ethical committees of the two universities involved.

The study was conducted in three phases. The first phase encompassed the initial development of the scale. Based on the results from focus group discussions with violence-affected women in northern Iraq identifying acts and patterns of IPV relevant to their living contexts, a panel of clinical experts arranged the resulting IPV items into four categories. In the second phase, data on IPV exposure and psychopathology were collected among a sample of 1,009 Iraqi and Syrian displaced women. The third phase consisted of the statistical analysis to assess the psychometric properties of the scale using confirmatory factor analysis (CFA), measuring the prevalence of violence exposure and mental health impairment among the participants, and determining the scale’s convergent validity.

### Phase 1: Development of the IPV Instrument

#### Item Generation

The first step of the development of the instrument was the generation of suitable items. Two focus group discussions with displaced Iraqi and Syrian women were conducted to discuss IPV acts and themes with the aim to incorporate types of violence into the proposed measure that are relevant for populations of women living in socially strained societies with high levels of gender inequality. This approach sought to increase the research’s local relevance following recommendations for gender-based violence research methods in humanitarian settings proposed by Hossain and McAlpine (2017). Each focus group consisted of 12 women residing in a refugee camp in the KRI who had been invited to participate through oral invitations by camp community mobilizers. The group discussions were held by a local female social worker specialized in working with women affected by violence. Using example items from pre-existing IPV scales such as the WHO Violence Against Women Instrument (Nybergh et al., 2013) and the Composite Abuse Scale (Hegarty et al., 1999, 2005), the participating women were asked about acts of partner violence that play a role in their own lives or within their communities.

After in-depth consultation with local experts in violence research, all IPV acts identified by the focus group participants were assessed for face validity by three members of the research team, as well as for their alignment with recommendations and guidelines for domestic violence research (WHO, 2001; Ellsberg and Heise, 2005; Hossain and McAlpine, 2017) to determine their inclusion in the questionnaire. That resulted in a list of 23 items covering acts of physical, emotional, verbal, sexual, controlling, and economic abuse. Since the focus group discussions had been conducted in Kurdish and Arabic languages, the generated item list was translated to English for further analyses. All translations including those described below were performed by multilingual clinical experts following translation guidelines for transcultural research (Human Services Research Institute, 2005).

#### Item Categorization

The next step of the process was to prepare the item list for psychometrically evaluation among Iraqi and Syrian displaced couples. A panel of six local and international clinical experts in violence research organized the identified 23 items based on patterns identified by the women in the focus group discussions and based on theoretical considerations of the content, meaning, and motivational characteristics of the acts within the given context of gendered societal norms of the Middle East. That resulted in a typology classifying violent acts against women in partnerships into four categories that were labeled as *Dominating behaviors*, *Existential threats*, *Impulsive aggressions (physical and verbal)*, and *Aggravated physical assault*.

Assigned to the category of *Dominating behaviors* were those acts of IPV which had been described by women in the focus groups as reflecting the husband’s intention to control, such as violating their freedom through deprivation of rights, interdictions, and coercive sexual acts. Seven items were identified to be fitting to these criteria, namely: (1) Being followed or watched, (2) being prevented from visits to family or friends, (3) controlled clothing decisions, (4) being prevented from working/studying, (5) forced sexual intercourse, (6) disregard during sexual intercourse, and (7) forceful impregnation.

Assigned to the *Existential threats* category were those IPV behaviors which, while they are also closely related to the subordination of women and their forced obedience to a male partner, were described as potentially posing severe risk of losing status and of social disadvantage. The items included in this subscale are all more or less economic and finance-related, such as being denied access to financial means or being forced to sell one’s possessions. Six items were included in this category: (8) Threat to get another wife/partner beside the spouse, (9) threat of being divorced, (10) threat to be thrown out of the house, (11) being forced to ask family or friends for money, (12) being forced to sell own personal possessions, and (13) to be denied financial means even if they are available.

Items reflecting acts described as mainly impulsive and to be occurring during situational partner conflicts, such as yelling or throwing things, were assigned to the *Impulsive aggressions* category. The six items assigned to this category were (14) name-calling, (15) use of disrespectful language, (16) pushing, hitting, kicking, beating, punching, slapping, (17) pulling the hair, (18) twisting arms, and (19) throwing things at the partner.

Finally, the category of *Aggravated physical assault* comprises IPV acts of intense physical violence with potential health- and life-threatening consequences (e.g., burning, attacks with weapons, etc.). This category had four items assigned to it: (20) Strangulation/attempting to strangle the partner, (21) burning or scalding, (22) attempt to kill the partner with a weapon, and (23) attacking the partner with a weapon/gun or knife.

The instrument was conceptualized as a checklist, as the aim of the study was to develop a short and pragmatic IPV instrument that is applicable in a variety of social contexts including complex humanitarian settings. However, in order to allow comprehensive assessments of both types and frequencies of IPV among this study’s participants, the preliminary 23-item questionnaire featured an answer format using a five-point-scale (scoring 0–4), indicating the frequency of the occurrence of each act with regard to the past year (*never*, *once*, *once per month*, *once per week*, or *daily*).

### Phase 2: Data Collection

#### Participants’ Characteristics

The sample recruited for the statistical analyses of this study consisted of 1,009 married Syrian (48.4%) and Iraqi (51.5%) women. Participants’ ages ranged from 15 to 75 years (*M* = 33.58, *SD* = 11.42) and the mean age for getting married was 19.43 (*SD* = 4.41, range 7–41). Almost all of the participating women were currently married (97.2%), while the other participants were either widowed (1.9%), divorced (0.5%), or separated (0.4%), though all participants had been with their partner within the past year at the time of assessment. The average duration of formal education was 4.26 years (*SD* = 4.32, range 0–18). Very few (6.4%) participants had an income of their own: 10,327.65 Iraqi Dinar (less than 8 EUR or 9 USD; *SD* = 65,479.04, range 0–900,000 Iraqi Dinar) per month on average. The women had 3.8 children on average (*SD* = 2.72, range 0–15); less than a tenth (8.9%) had no children at all.

#### Procedures and Instruments

Data collection was conducted in camps for displaced people located in Duhok and Sulaymaniyah, KRI. Due to lack of reliable census data for the camps, sampling was performed using a pragmatic approach. The camps were subdivided into sections according to approximately equal household numbers. Households in each section were randomly selected for participation by spinning a pen from the section center on a camp map. Interview staff then visited the selected households to determine eligibility of women for the study. Approval of the study procedures was provided by the camp administrations and the ethical committees of Bielefeld University, Germany, and Koya University, KRI. Structured interviews were conducted with participants in either Arabic (41.7%) or Kurdish (58.3%). Interviews lasted between 60 and 90 min and took place in the homes of the participants without any other person present to insure privacy and confidentiality. All interviewers were fluent in Kurdish and Arabic, held University degrees in either psychology or social work, and had been trained in data collection procedures prior to conducting assessments. Due to cultural considerations, participants’ consent was obtained in verbal rather than written form after informing them about the study’s procedures and their rights as participants (Ibrahim and Hassan, 2017). For underage participants, their parents’ consent was additionally obtained. A comprehensive risk management procedure was established to protect participants and staff. Women who reported being affected by severe violence or mental health issues were offered counseling by psychologically trained staff and were referred to further health care providers if needed. Telephone numbers of emergency and violence prevention hotlines and contacts to local support organizations in and outside of camps were also handed out to participating women. Details on the comprehensive measures taken to protect respondents and staff during and around data collection, including the focus groups, to ensure ethically sound research procedures have been described in previous publications generated from this research project (Ibrahim et al., 2018a,b, 2019; Goessmann et al., 2019).

As the present study focuses on violence against women in partnerships, it includes only data from women. In addition to collecting information on participants’ experiences of IPV, sociodemographic information and mental health issues, in terms of depression and posttraumatic stress disorder (PTSD), were also assessed using validated instruments. PTSD symptoms were measured using the Arab and Kurdish versions of the PTSD Checklist for DSM-5 (PCL-5) which had been validated in the KRI (Ibrahim et al., 2018a). Depression symptoms were measured with the 15-item depression subscale of the Hopkins Symptom Checklist (HSCL-D), a valid cross-cultural instrument that has been utilized previously in displaced Arabic populations (Tinghög and Carstensen, 2010; Al-Turkait et al., 2011). Internal consistency of both the PCL-5 and HSCL-D in this sample was good, as indicated by Cronbach’s alpha values of α = 0.91 (PCL-5) and α = 0.86 (HSCL-D), respectively.

### Phase 3: Statistical Analysis

**FIGURE 1:**
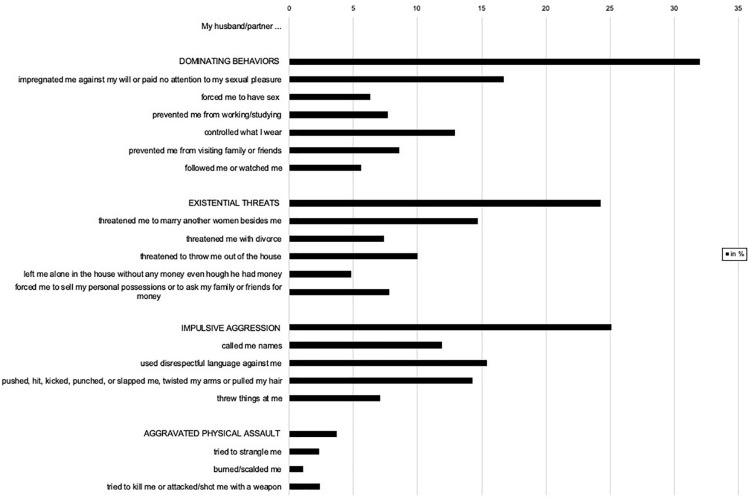
Past-year male-perpetrated partner violence reported by women (*N* = 1009).

Means, standard deviations, ranges, and frequencies were calculated to describe the sample characteristics as well as violence and psychopathology prevalence. To examine the proposed item structure of the Gendered Violence in Partnerships Scale (GVPS), CFA was performed. Model fit of the CFA was tested along the criteria for model fit indices suggested by Schermelleh-Engel et al. (2003). Since the Chi-square test is extremely sensitive to large sample sizes (Bentler and Bonett, 1980), we instead relied on other fit indices including Comparative Fit Index (CFI), Tucker–Lewis Index (TLI), Incremental Fit Index (IFI), Root-Mean-Square Error of Approximation (RMSEA), and Standardized Root-Mean-Square Residual (SRMR). Model fit was considered to be acceptable if the model featured TLI, IFI, and CFI values of ≥0.90, an RMSEA value of ≤0.06 with 90% confidence interval values <0.05 (lower value) and <0.08 (upper value), and an SRMR value below 0.08. In the first step of the CFA, the 23 continuous items of the preliminary questionnaire were entered according to the four preassigned subscales (Table 1). After an initial examination of the results, the item loadings and the scale structure were discussed by a panel of transcultural clinical experts. The discussion resulted in recoding and rearranging of the items based on similarity and co-occurrence of items and on contextually informed considerations to reflect the participating women’s lived realities. Then, the CFA was run again. The final scale resulting from the second step of the CFA was then used to calculate descriptive statistics of the participants’ IPV experiences (see Figure 1), as well as indicators of internal consistency and convergent validity based on associations with mental health measures (see Table 3). Data analyses were carried out using IBM SPSS and Amos version 25. The internal consistency of the resulting IPV scale and the subscales was tested using Cronbach’s alpha reliability. Convergent validity was measured on the basis of correlation analyses of the IPV sum score and the IPV subscale scores with mental health indicators (depression and PTSD scores).

**TABLE 1:** Factor loadings of step 1 of the factor analysis including 23 items.

Item	Factor 1	Factor 2	Factor 3	Factor 4
Has your partner impregnated you against your will?^1^	0.350			
Has your partner disregarded you during sex and only focused on their own pleasure?^1^	0.335			
Has your partner forced you to have sex when you did not want to?	0.622			
Has your partner prevented you from working/studying?	0.447			
Has your partner controlled what you wear?	0.531			
Has your partner prevented you from visiting your family or friends?	0.655			
Has your partner followed you or watched you?	0.634			
Has your partner left you alone in the house without money even though they had money?		0.596		
Has your partner forced you to sell your personal possessions (e.g., house or jewelry)?^2^		0.707		
Has your partner forced you to ask your family or friends for money?^2^		0.532		
Has your partner threatened to throw you out of the house?		0.830		
Has your partner threatened you with divorce?		0.783		
Has your partner threatened to get another wife/partner?		0.605		
Has your partner thrown things at you?			0.770	
Has your partner twisted your arms?^3^			0.826	
Has your partner pulled your hair?^3^			0.840	
Has your partner pushed, hit, kicked, beaten, punched, or slapped you?^3^			0.824	
Has your partner used disrespectful language toward you?			0.750	
Has your partner called you names?			0.738	
Has your partner attacked you with a weapon (such as a gun or a knife)?^4^				0.361
Has your partner tried to kill you with a weapon?^4^				0.488
Has your partner burned or scalded you?				0.742
Has your partner tried to strangle you?				0.927

*Model fit: χ^2^[224, *N* = 1009] = 1996.66 (*p* < 0.001), CFI = 0.85, RMSEA = 0.089 [90%-CI = 0.085 −0.092, PCLOSE = 0.00], SRMR = 0.06.*

*^1^Two items which were combined into one item on sexual subjection in step 2 of the CFA.*

*^2^Two items that were combined into one item on forced money acquisition in step 2 of the CFA.*

*^3^Three items that were combined into one item on physical attacks in step 2 of the CFA.*

*^4^Two items that were combined into one item on physical attacks with weapons in step 2 of the CFA.*

## Results

### Confirmatory Factor Analysis of the Gendered Violence in Partnerships Scale

The first step of the CFA included all 23 items initially derived from the focus group discussions and resulted in a model with inadequate fit (CFI = 0.85, RMSEA = 0.089 [90%-CI = 0.085 −0.092, PCLOSE = 0.00], SRMR = 0.06). Some items showed very low factor loadings (i.e., below 0.40; see Table 1). After discussing the results with a group of experts, a re-arrangement of the items was made with the main aim not to lose any informative value of the initial item list. Since the items with low factors loadings represented rather rare events but were nonetheless reflective of relevant experiences of the women participating in the focus groups (e.g., “Has your partner tried to kill you with a gun?”), their information was retained.

In step 2 of the factor analysis, instead of excluding items with lower factor loadings, thematically related items were combined and rephrased to create four new items (see Table 2). This led to the reduction of the total number of items from 23 to 18. In the subscale of *Dominating behaviors*, two items addressing sexual violence (having one’s own sexual needs ignored by their partner; getting impregnated against their will) were combined to create one new item of sexual subjugation. The two items regarding the forced acquisition of money (being forced to ask family or friends for money; being forced to sell one’s personal possessions) were combined into one new item under the subscale of *Existential threats*. The three items representing physical attacks without objects (being pushed/kicked/slapped; having their hair pulled; having their arms twisted) were combined into one item in the subscale of *Impulsive aggressions*. Lastly, in the *Aggravated physical assault* subscale, the two items addressing physical violence with weapons (attempted murder with a weapon; attack with a gun or knife) were combined into one item. The number of items in each of the four subscales (*Dominating behaviors*, *Existential threats*, *Impulsive aggressions*, and *Aggravated physical assault*) were reduced to six, five, four, and three items, respectively (see Table 2). Since the study aimed for the creation of a pragmatic IPV assessment instrument to be used in unstable contexts such as displacement camps where the feasibility of extensive surveys is limited, the scale’s response format was changed to a binary format (no/yes; scored 0–1). In order to provide complete versions of the instrument in the languages in which the original items were generated, the four combined items were back-translated to Arabic, Kurdish Kurmanji, and Kurdish Sorani. Translations were performed by clinical experts with experience in instrument translations, and translation accuracy was verified by independent language experts. The adaptations of step 2 resulted in a checklist of 18 items with acceptable model fit (CFI = 0.93, TLI = 0.91, IFI = 0.93, RMSEA = 0.055 [90%-CI = 0.05 −0.06, PCLOSE = 0.06], SRMR = 0.04) and moderate to high factor loadings between 0.40 and 0.80 (see Table 2). The resulting GVPS was then used to determine participants’ overall IPV prevalence and subscale scores (see below and in Figure 1).

**TABLE 2:** Factor loadings of the final factor solution of the GVPS including 18 binary-coded items.

Item (new item number in 18-item checklist)	Factor 1	Factor 2	Factor 3	Factor 4
**Has your partner impregnated you against your will or has your partner neglected you during sex and only focused on their own pleasure? (sexual subjugation)**	0.433			
Has your partner forced you to have sex when you did not want to?	0.583			
Has your partner prevented you from working/studying?	0.406			
Has your partner controlled what you wear?	0.475			
Has your partner prevented you from visiting your family or friends?	0.558			
Has your partner followed or watched you?	0.525			
Has your partner left you alone in the house without any money even though they had money?		0.509		
**Has your partner forced you to sell your personal possessions (e.g., house or jewelry) or forced you to ask your family or friends for money?**		0.569		
Has your partner threatened to throw you out of the house?		0.704		
Has your partner threatened you with divorce?		0.664		
Has your partner threatened to get another wife/partner?		0.553		
Has your partner thrown things at you?			0.688	
**Has your partner pushed, hit, kicked, beaten, punched, or slapped you, twisted your arms or pulled your hair?**			0.722	
Has your partner used disrespectful language toward you?			0.695	
Has your partner called you names?			0.664	
**Has your partner tried to kill you or attacked you with a weapon, gun or knife?**				0.633
Has your partner burned or scalded you?				0.598
Has your partner tried to strangle you?				0.800

*Model fit: χ^2^[129, *N* = 1009] = 518.07 (*p* < 0.001), CFI = 0.93, TLI = 0.91, IFI = 0.93, RMSEA = 0.055 [90%-CI = 0.05 –0.06, PCLOSE = 0.06], SRMR = 0.04. New items obtained through combinations of previous items are in bold.*

**TABLE 3:** Bivariate correlations between IPV scores and mental health scores.

	1	2	3	4	5	6	7	8	9	10	11
1 IPV CL sum score	__	0.86**	0.65**	0.89**	0.85**	0.22**	0.29**	0.23**	0.20**	0.24**	0.28**
2 Subscale Impulsive aggressions (physical/verbal)		__	0.48**	0.70**	0.58**	0.19**	0.26**	0.22**	0.16**	0.21**	0.25**
3 Subscale Aggravated physical assault			__	0.53**	0.51**	0.13**	0.17**	0.11**	0.16**	0.17**	0.15**
4 Subscale Existential threats				__	0.64**	0.21**	0.27**	0.23**	0.19**	0.22**	0.27**
5 Subscale Dominating behaviors					__	0.18**	0.26**	0.18**	0.15**	0.18**	0.22**
6 HSCL-D sum score						__	0.68**	0.54**	0.30**	0.64**	0.65**
7 PCL-5 sum score							__	0.85**	0.57**	0.90**	0.88**
8 PCL-5 Intrusions								__	0.39**	0.67**	0.65**
9 PCL-5 Avoidance									__	0.41**	0.41**
10 PCL-5 Cognitions and mood										__	0.71**
11 PCL-5 Arousal											__

Pearson’s correlations, two-tailed. HSCL-D, Hopkins Symptom Checklist for Depression; PCL-5, PTSD checklist for DSM-5. **p < 0.01.

### IPV Exposure and Psychopathology

Experience of IPV was high among the interviewed women, with 442 (43.8%) reporting that they had experienced at least one violent act perpetrated by their partner within the past year. The most common type of IPV reported was *Dominating behaviors* (reported by 32%), followed by *Impulsive aggressions* (25.1%), *Existential threats* (24.3%), and *Aggravated physical assault* (3.7%). Specific acts of IPV that were reported by more than 10% of the participating women included control of clothing, denial of sexual and reproductive rights, threats to get another wife, threats to be thrown out, being called names, disrespectful language use, and physical attacks (hitting, kicking, twisting arms, pulling hair). Frequencies of all individual acts and subtypes of IPV reported by the participants can be found in Figure 1. Psychopathology was high, with 72% of the participants (*M* = 35.32, *SD* = 17.74) endorsing PTSD symptom levels above the adapted PCL-5 cut-off value of 23 (Ibrahim et al., 2018a). An even larger proportion of participants (81.9%; *M* = 2.23, *SD* = 0.76) endorsed clinically relevant levels of depressive symptoms as measured by the HSCL-D (score > 1.55).

### Reliability and Validity

The full GVPS, as well as its four subscales, showed moderate to good internal consistency indicated by Cronbach’s alpha reliability values between 0.65 and 0.88. For the *Dominating behaviors* subscale reliability was α = 0.65, for the *Existential threats* subscale α = 0.72, for the *Impulsive aggressions* subscale α = 0.78, for the *Aggravated physical assaults* subscale α = 0.70, and for the full scale it was α = 0.88. The four subscales correlated significantly with each other and with the sum score. Correlation coefficients ranged from 0.48 to 0.89. The subscale *Aggravated physical assaults* showed the lowest correlations with the other subscales as well as with the GVPS sum score (*r* = 0.65, *p* < 0.01), while the correlations of the three other subscales with the GVPS sum score were all well above 0.80 (see Table 3).

The total score as well as the subscale scores of the GVPS showed good convergent validity with measurements of women’s mental health status. All measures of PTSD and depression symptoms were significantly correlated with the GVPS score and the four subscale scores (see Table 3). Correlations with depression were similarly high for experiences of *Existential threats* (*r* = 0.21, *p* < 0.01), *Impulsive aggressions* (*r* = 0.19, *p* < 0.01) and *Dominating behaviors* (*r* = 0.18, *p* < 0.01), and lowest for experiences of *Aggregated physical assaults* (*r* = 0.13, *p* < 0.01). The correlations of *Existential threats* and *Impulsive aggressions* with depression were both significantly higher than the correlation of *Aggregated physical assaults* with depression, *Z* = −2.67, *p* < 0.01 and *Z* = −1.90, *p* < 0.05. All other subscale correlations with depression did not differ significantly from each other. The pattern was similar for PTSD symptoms, with *Existential threats*, *Impulsive aggressions* and *Dominating behaviors* all showing significant correlations above 0.26 with the PCL-5 sum score. The correlation of PTSD with *Aggregated physical assaults* (*r* = 0.17, *p* < 0.01) was significantly smaller than the correlation of PTSD with *Existential threats* (*Z* = −3.28, *p* < 0.001), *Impulsive aggressions* (*Z* = −2.89, *p* < 0.01), and *Dominating behaviors* (*Z* = −2.97, *p* < 0.001).

## Discussion

The present study described the development process and psychometric evaluation of the GVPS (see Table 4), a new checklist for the assessment of IPV, that was evaluated in a displacement setting in the Middle East. The study fills a gap in the literature of adequate IPV assessment for women in violent-torn environments by providing a pragmatic, contextually valid event checklist. The primary aim of the study’s three-phase development procedure was to ensure the process to be locally informed in order to create a pragmatic instrument that reflected the living situations of the women involved. To this end, focus groups of Syrian and Iraqi displaced women discussed and identified acts and patterns of IPV prevalent in their community. The emerging IPV items were then checked for face validity by local and international experts and arranged into four thematic categories, and the resulting item list was psychometrically analyzed using the data from 1,009 Syrian and Iraqi displaced women.

**TABLE 4:** Gendered violence in partnerships scale (GVPS) (English version).

	No (0)	Yes (1)
Dominating behaviors		
Has your partner followed you or watched you?		
Has your partner controlled what you wear?		
Has your partner prevented you from visiting your family or friends?		
Has your partner prevented you from working/studying?		
Has your partner forced you to have sex when you did not want to?		
Has your partner impregnated you against your will or has your partner neglected you during sex and only focused on his own pleasure? (sexual subjugation)		
Existential threats		
Has your partner threatened you with divorce?		
Has your partner threatened to throw you out of the house?		
Has your partner forced you to sell your personal possessions (e.g., house or jewelry) or forced you to ask your family or friends for money?		
Has your partner left you alone in the house without any money even though they had money?		
Has your partner threatened to get another wife/partner?		
Impulsive aggressions (verbal and physical)		
Has your partner called you names?		
Has your partner pushed, hit, kicked, beaten, punched, or slapped you, twisted your arms or pulled your hair?		
Has your partner thrown things at you?		
Has your partner used disrespectful language toward you?		
Aggravated physical assault		
Has your partner tried to strangle you?		
Has your partner tried to kill you or attacked you with a weapon, gun or knife?		
Has your partner burned or scalded you?		

*Versions of the GVPS in Arabic, Kurdish Kurmanji, and Kurdish Sorani are available upon request.*

The results of the study provide evidence for the validity and reliability of the GVPS. A two-step factor analysis confirmed the scale’s psychometric properties and its proposed factor structure of *Dominating behaviors*, *Existential threats*, *Impulsive aggressions*, and *Aggregated physical assault*. While in the first step of the factor analysis, the initial model with 23 items showed inadequate model fit and some critically low factor loadings, the model fit of the 18-item version was acceptable, with moderate to high factor loadings on all four subscales. Its model fit indices are in line with other measurement scales evaluated using CFA (e.g., Boduszek et al., 2018; Hooker et al., 2019). Somewhat lower but still acceptable factor loadings between 0.40 and 0.50 were observed for three items in the *Dominating behaviors* subscale, which also had the lowest Cronbach’s alpha coefficient (α = 0.65). This finding might best be explained by the inclusion of both sexually and non-sexually dominating acts in this subscale. The reason for the integration of those different acts into one subscale was based on the supposition that their common elements were their oppressive nature and the manner in which they put women in a position of subordination under a male partner’s control and domination (Kelly and Johnson, 2008). Research has indicated that sexual coercion, as well as psychological control and intimidation, increase negative health outcomes for women, especially if they co-occur, which highlights the role of gender and power relations for the impacts of IPV (Pico-Alfonso et al., 2006; Caldwell et al., 2012). The item with the lowest scoring on the *Dominating behaviors* subscale was the prevention from working or studying. In previous IPV instruments, this behavior has been assigned to acts of economic oppression (Adams et al., 2008), which are represented in our *Existential Threats* subscale. However, discussions with local experts revealed that, in the given social context, being denied access to education or work is considered an act of control rather than an existential threat, since men’s intention to regulate women’s every behavior and whereabouts is the driving underlying motivation for it. The *Dominating behaviors* subscale thus makes theoretical sense in the context, and its internal consistency of α = 0.65 can be considered acceptable for a subscale with six items covering two different aspects (i.e., psychological and sexual acts) of controlling and dominating behaviors (Streiner, 2003).

The four established subscales correlated significantly with each other as well as with the GVPS sum score. Only the correlations of the *Aggravated physical assault* subscale were somewhat lower than those between the other three subscales. A possible explanation for this finding might be the relative rareness of the events covered by the three items of the *Aggravated physical assault* subscale (i.e., strangulation; burning/scalding; attacks with weapons), which limits its representativeness for the GVPS as a whole and enhances skewness of the distributions. Despite their comparatively rare report, collecting information on acts of extreme physical violence is crucial to understand the full extent of the variety of women’s IPV experiences. It is important to keep in mind that more severely abused women tend to be less likely to report their abuse or participate in surveys, and reaching them might require particular efforts (Waltermaurer et al., 2003). That may also explain the lower reported frequencies of the *Aggravated physical assault* items, a finding for which possible reporting biases should also be considered responsible due to potential fear and shame. By contrast, *Dominating behaviors* were the most prevalent forms of violence reported in this sample, indicating that acts of manipulation and control, as well as sexual coercion, are common experiences in the daily lives of many of the participating women. The frequent report of events such as having their sexual and reproductive rights denied or clothing regulations draws a dark picture of the subordination of women and highlights the patriarchal contexts in which IPV often occurs. The belief that holds women to be inferior to men is still prevalent across the globe. Women’s rights are disrespected in many ways, and women are often expected to subordinate themselves, which in turn can facilitate their victimization of physical violence (Namy et al., 2017). The high correlations of both the *Existential threats* and the *Dominating behaviors* subscales with the *Impulsive aggressions* subscale, which showed particularly high prevalence for the items on physical violence and disrespectful language use, also indicated the connection of subordination and physical violence victimization. Overall, the prevalence of 44% found in this sample for the whole GVPS exceeds the average prevalence level of 35% previously reported for Middle Eastern countries (Devries et al., 2013) and shows partner violence, in its numerous forms, to be a significant issue among displaced Syrian and Iraqi couples. This is in line with previous research indicating burdened and violent partnership and family relations in conflict-affected contexts (Catani, 2010; Stark and Ager, 2011). However, in light of frequent underreporting of IPV, especially in cases where the relationship with the abuser is ongoing and if women themselves tend to justify spousal violence (Al-Modallal, 2015), it has to be kept in mind that this number might still be an underestimation of the actual severity of abuse experienced by the interviewed women.

The study’s results further provide initial indications of good convergent validity of the GVPS and its subscales. Significant correlations of IPV with measures of depression and PTSD symptomatology were found, which is in line with previous research highlighting the negative impacts of IPV on women’s mental health (Ellsberg and Emmelin, 2014). Some of the highest correlations of psychopathology measures with subtypes of IPV were those which are a product of male dominance over women (i.e., dominating behaviors and existential threats). This sheds light on the often neglected living situation of Iraqi and Syrian women in the KRI, which seems to be characterized by an intertwining of health impairment and ongoing violence embedded in patriarchal societal structures (Johnson and Leone, 2005). Elucidating the dynamics of the home context of Iraqi and Syrian women seems critical, as the effects of psychologically manipulative acts on (mental) health often go unnoticed, particularly if they occur in combination with other forms of IPV (Arriaga and Schkeryantz, 2015). Existential threats in particular bear the risk of holding the affected women in a continuous state of helplessness, as possibilities to seek support are limited by the abuse itself, for example when contact with friends and relatives is forbidden.

The present study has some important implications for theoretical IPV research and practice as well as for intervention efforts to improve the living conditions of violent-affected women. Following the call by scholars and practitioners in the field to develop instruments differentiating between thematic types of IPV and to empirically validate them in different settings and populations (Kelly and Johnson, 2008; Ali et al., 2016), this study is a first step toward these goals providing an instrument with new meaningful IPV categories that go beyond a categorization of IPV into physical, psychological and sexual abuse types. Assessing IPV experiences clustered in patterns of dominating behaviors, existential threats, impulsive aggressions, and aggravated physical assaults might be useful to professionals to detect underlying dynamics and thus tailor specified interventions. Furthermore, discussing those patterns with violence-affected women might help them gain awareness for indications and associations of IPV in their partnerships. The results on frequency levels and mental health associations found in this study are indications of the value of the proposed GVPS subscales for comprehensive assessments of the prevalence and manifestations of IPV in a setting of predominant gender hierarchies. The high level of IPV exposure, as well as its correlations with depression and PTSD psychopathology found in this study, indicate ongoing insecurity and hardship for women living in (post-)war contexts, an issue that calls for focused attention and action within humanitarian care efforts. Although we developed the scale in a specific context, and the test of the GVPS in other social and cultural settings is still pending, the GVPS items cover a variety of violent acts potentially relevant to the lived realities of many women worldwide and thus offer possibilities to investigate conditions and circumstances of IPV. Future usage and application of the GVPS in different settings should demonstrate that the new categorization provided by this instrument is helpful in the study of the causes and consequences of IPV and may thus help gather further details on the circumstances of violence against women in partnerships to plan and conduct interventions appropriately.

### Strengths and Limitations

To our knowledge, this is the first study to scientifically develop and validate an IPV instrument among a population of displaced women in the Middle East using a pragmatic approach with focus groups and expert group discussions, including members of the local communities. As opposed to previous developments or adaptations of IPV scales, our procedure followed a bottom-up approach using the experiences and perspectives of the target population as a starting point for the scale development. This approach enabled the creation of an instrument that takes the lived realities of violence-affected women into account by actively engaging them in the process. The collaboration of international and local clinical experts in the scale development further enhanced the adoption of multiple perspectives in the process and dismantled popular prerogatives of interpretation (Webb, 1993; Hossain and McAlpine, 2017).

Further, the project’s realization by an international team and within Arab and Kurdish speaking areas made the simultaneous development of the scale in four languages possible; thus, the instrument is now available in English, Arabic, Kurdish Kurmanji, and Kurdish Sorani. Another advantage of the study is the size of the sample used for data collection. The sample is a good representation of women living in a setting of gender inequality and daily struggle due to ongoing social and political instabilities. However, it has to be noted that some specific characteristics of the participating displaced women might impact their IPV levels. Thus, their experiences do not necessarily reflect the lived realities of other women in Syria, Iraq, or elsewhere. For example, some of the items, such as the threat of getting another wife, are highly context-dependent. The *ad hoc* development of the scale’s subscales reflects the study’s pragmatic approach to create a contextually valid IPV instrument; however, it might limit the scale’s generalizability across social and cultural contexts. The present study demonstrated the scale’s suitability and utility in a post-war environment in the Middle East. However, the item categorization and subscales of the GVPS was based on IPV patterns identified as prevalent in the local population and might not be transferable to other contexts. Thus, the applicability, factor structure, and validity of the GVPS need to be tested by future studies in other contexts in order to prevent the risk of premature and inappropriate cross-cultural generalizations (Clark and Walker, 2011), and to make broader analyses of types and circumstances of IPV possible.

Furthermore, the external validity of the scale was tested only by using associations to women’s mental health outcomes. Other validity measures, such as future predictive validity could not be assessed here due to the study’s design. Future studies should investigate broader associations of IPV, including perpetrators’ characteristics, as well as the question of recidivism of different IPV types to identify risk factors and promote prevention. The GVPS provides a promising tool for such analyses in longitudinal designs.

## Conclusion

The present study introduces the GVPS, a new event checklist to assess experiences of IPV against women that was developed among women from displaced communities in northern Iraq. The development process followed a pragmatic approach aiming to increase local validity of the resulting scale by directly involving local communities and clinical experts who discussed themes and events of IPV against women in the social and cultural context. Statistical analysis found indicators of good psychometric properties of the scale and confirmatory factor analyses of the item structure confirmed the typology of four thematic subscales of IPV to be reflective of the involved women’s living situations (*Dominating behaviors*, *Existential threats*, *Impulsive aggressions*, and *Aggregated physical assault*). Furthermore, the study’s findings on IPV prevalence and associations with psychopathology significantly extend existing knowledge about IPV and its impacts in settings with high levels of social and political challenges. This newly developed IPV assessment tool might help to understand theoretically the nature of violence and abuse against women in highly patriarchal societies by integrating notions of power relations in gender-based violence. Furthermore, it has the potential to enable health professionals to reliably and validly estimate the suffering that stems from IPV in Iraq, other Arab countries, and beyond in order to promote the development of adequate interventions combatting the global issue of gender-based violence.

## Data Availability Statement

The raw data supporting the conclusions of this article will be made available by the authors, without undue reservation.

## Ethics Statement

The studies involving human participants were reviewed and approved by Ethics committees of Bielefeld University, Bielefeld, Germany and Koya University, Koya, Iraq. Participants’ informed consent was obtained in oral form. After receiving detailed information on participation and the study’s objectives, each participant’s informed consent and agreement to participate was documented carefully by the interviewer on a standardized consent information form.

## Author Contributions

FN and HI obtained the funding of the research. KG was the main contributor of the specific design of this study. KG and HI managed and supervised to collect the data. HI, LS, and KG performed the data analysis. FN, LS, and HI contributed to data interpretation and the structure of the manuscript. KG drafted the manuscript and all authors contributed to the final version.

## Conflict of Interest

The authors declare that the research was conducted in the absence of any commercial or financial relationships that could be construed as a potential conflict of interest.
